# Inflammation Beats Cholesterol: A Comment on the Unequivocal Driver of Cardiovascular Disease Risk

**DOI:** 10.3390/jcm12072519

**Published:** 2023-03-27

**Authors:** Mauro Vaccarezza, Francesco Maria Galassi

**Affiliations:** 1 Curtin Medical School, Faculty of Health Sciences, Curtin University, Bentley, Perth, WA 6102, Australia; 2 Curtin Health Innovation Research Institute, Faculty of Health Sciences, Curtin University, Bentley, Perth, WA 6102, Australia; 3 Department of Environmental and Prevention Sciences, University of Ferrara, 44121 Ferrara, Italy; 4 Department of Anthropology, Faculty of Biology and Environmental Protection, University of Lodz, 90-237 Lodz, Poland; francesco.galassi@biol.uni.lodz.pl

**Keywords:** inflammation, atherosclerosis, cardiovascular risk, statins

## Abstract

Despite advancements in the current standard of care, cardiovascular diseases continue to hold the top spot as the leading cause of mortality worldwide. The development of atherosclerosis is the most common culprit behind ailments such as myocardial infarction, stroke, and peripheral vascular disease. Consequently, it imposes a significant burden on life expectancy, quality of life, morbidity, and societal costs. Both increased cholesterol levels and the activation of the inflammatory cascade are known as cardiovascular risk facts. Their relative weight is in the spotlight of curent biomedical research. Newly published data shed light on the role of inflammation in determining cardiovascular risk irrespective of cholesterol levels and cholesterol-lowering therapies.

## 1. Introduction

Cardiovascular disease (CV) is one of the most ancient forms of pathology in *Homo sapiens* dating back to the most ancestral societies; currently, it is peaking in our times because longer life spans are made possible by improved hygiene and sanitation and increased food supplies, especially in the Western world, hence allowing people to reach the fourth to seventh decade of life, in which such chronic ailments become more phenotypically evident [1,2,3,4,5]. Remarkably, in atherosclerosis, the deposition of cholesterol, lipids, and inflammatory events in specific areas of blood vessels trigger endothelial damage and the proliferation of smooth muscle cells. Overall, the pathogenesis of atherosclerosis encompasses oxidative stress, high levels of oxidized lipoproteins, immune reactivity, and inflammation [6]. Moreover, hypercaloric diets and stressful lifestyles in the Western world make this category of disease rampant. The resulting obesity epidemic increases CV risk, considering the substantial reduction of plasma cholesterol clearance ability in obese subjects [7]. Despite medical advances, CV is a leading cause of death worldwide, and it is estimated that over 17 million people die from it each year [8]. Such figures are staggering if one considers that it is about the same number of people who died in World War One. Statins are a commonly prescribed medication used to lower cholesterol levels and reduce the risk of cardiovascular events. However, recent studies only proved and emphasized the significance of the role of inflammation in cardiovascular risk [9,10,11,12,13,14]. Furthermore, inflammation is a pivotal player not only as a systemic condition but also as a substantial determinant of CV risk in localized settings such as the oral cavity [15].

## 2. The Link between Inflammation and Cardiovascular Risk

Inflammation is a natural response of the body’s immune system to injury or infection. However, chronic inflammation can lead to the development of atherosclerosis, a condition where plaque builds up in the arteries and restricts blood flow. This can increase the risk of heart attack and stroke [12,13,14,16]. Inflammation can also cause damage to the endothelium, the inner lining of blood vessels, which can lead to the formation of blood clots. Studies have shown that inflammation markers such as high-sensitivity C-reactive protein (hs-CRP) and interleukin-6 (IL-6) are elevated in patients with cardiovascular disease. A plethora of soluble mediators is involved in determining this pro-inflammatory milieu [16,17,18]. Of note, IL-6, together with other pro-inflammatory cytokines such as Tumor Necrosis Factor-alpha (TNFa) and Interleukin-1 (IL-1), can inhibit triglyceride synthesis and, at the same time, can promote insulin resistance in various target tissue, highlighting the complex interplay between metabolic and biochemical inflammatory pathways in CV [19]. These markers have also been found to be elevated in patients receiving statin therapy, despite their cholesterol levels being well controlled [9,11,12,13,14,15,16,17]. This scenario suggests that inflammation may indeed play a significant role in cardiovascular risk, even in patients who are already taking statins [11,12,13,14,15,16,17].

### 2.1. The Role of Statins in Reducing Inflammation

Statins work by inhibiting the enzyme β-Hydroxy β-methylglutaryl-CoA (HMG-CoA, a metabolic intermediate in branched-chain amino acids metabolism) reductase, which is involved in the production of cholesterol. However, they also have anti-inflammatory properties. Statins have been shown to reduce the levels of hs-CRP and IL-6 in patients with cardiovascular disease, suggesting that they may help to reduce inflammation and lower cardiovascular risk [7,20,21,22,23].

### 2.2. The Importance of Monitoring Inflammation in Patients on Statin Therapy

While statins are effective at reducing cholesterol levels and lowering cardiovascular risk, it is important to monitor inflammation markers in patients receiving statin therapy. Elevated levels of hs-CRP and IL-6 may indicate that a patient is at increased risk of cardiovascular events, even if his cholesterol levels are well controlled. In these cases, additional therapies such as anti-inflammatory medications may be necessary to further reduce cardiovascular risk [22,23,24].

## 3. New Insights and Their Significance

Although statins are efficient in reducing low-density lipoprotein (LDL)-cholesterol concentration and decreasing cardiovascular risk, cardiovascular diseases are still a significant cause of death worldwide. This is due to the increasing prevalence of the so-called “civilization diseases” such as obesity, type 2 diabetes, metabolic syndrome, and lipid disorders. Additionally, studies show that inflammation plays a crucial role in the initiation, progression, and clinical manifestations of atherosclerosis [25,26]. Hence, it is essential to establish a clear understanding of the connection between inflammation and adverse clinical events related to atherosclerotic cardiovascular disease. Additionally, current and potential therapies that can be used in combination with statins to further lower lipids or reduce inflammation, as well as the relative contributions of cholesterol and inflammation to residual cardiovascular risk in patients undergoing statin therapy, need to be thoroughly examined.

In a recent report by Ridker et al., findings from a collaborative analysis evaluating the residual risks of cardiovascular events linked to elevated cholesterol and inflammation in statin-treated patients within a contemporary setting were shared [27]. The study was a meta-analysis of three major randomized trials, namely PROMINENT, REDUCE-IT, and STRENGTH, that included 31,245 patients (69.0% men, 31.0% women). The research aimed to compare the strength of the association between residual cholesterol risk and cardiovascular events/mortality with that of residual inflammatory risk as indicated by plasma high-sensitivity C-reactive protein (CRP) concentration. The results indicated that even though patients were receiving intensive statin therapy, residual inflammatory risk had a stronger correlation with cardiovascular events and mortality compared to residual cholesterol risk [27].

Higher concentrations of high-sensitivity CRP (highest high-sensitivity CRP quartile vs. lowest quartile) paralleled significantly with an increased risk of major adverse cardiovascular events (hazard ratio (HR) 1.31, 95% CI 1.20–1.43), cardiovascular mortality (2.68, 2.22–3.23), and all-cause mortality (2.42, 2.12–2.77) [26]. By contrast, the relationship of higher LDLC concentrations (residual cholesterol risk) was neutral for major adverse cardiovascular events (highest LDLC quartile vs. lowest quartile, HR 1.07, 95% CI 0.98–1.17) and of low magnitude for cardiovascular death (1.27, 1.07–1.50) and all-cause death (1.16, 1.03–1.32) [27].

Remarkably, the study’s extensive sample size provided ample statistical power to scrutinize correlations between biomarkers and cardiovascular outcomes. Despite several complexities pertaining to the employed prediction models and corresponding statistical analyses [28], this significant study implies that the residual risk of cardiovascular events and mortality is higher in association with inflammation than with cholesterol. This analysis revealed that residual inflammatory risk outweighs residual cholesterol risk because patients were already undergoing stringent statin therapy in the incorporated trials. Nonetheless, these findings hold significant clinical relevance for physicians when contemplating the addition of further lipid-lowering or inflammation-reducing therapies for patients receiving statin therapy.

As a matter of fact, healthcare professionals, specifically cardiologists and physicians, should shift their standpoint towards intensive lipid-lowering and inflammation-reduction therapies. These methodologies should no longer be regarded as mutually exclusive treatments but rather as complementary approaches for patients with atherosclerotic cardiovascular disease who are currently being treated with statin therapy.

## 4. Conclusions

Inflammation plays a significant role in cardiovascular risk, particularly in patients receiving statin therapy. While statins are effective at reducing cholesterol levels and lowering cardiovascular risk, they also have anti-inflammatory properties. It is important to monitor inflammation markers in patients on statin therapy to ensure that their cardiovascular risk is adequately managed. Additional therapies will be necessary for patients with elevated inflammation markers to further reduce their risk of cardiovascular events.

## Data Availability

Not applicable.
